# Abdominal incision defect following AAA-surgery (AIDA): 2-year results of prophylactic onlay-mesh augmentation in a multicentre, double-blind, randomised controlled trial

**DOI:** 10.1007/s13304-021-01125-0

**Published:** 2021-07-21

**Authors:** S. Honig, H. Diener, T. Kölbel, W. Reinpold, A. Zapf, E. Bibiza-Freiwald, E. S. Debus, for AIDA study group, Peter Breuer, Harald Daum, Hans-Henning Eckstein, Johannes Gahlen, Jochen Grommes, Thomas Hupp, Richard Kellersmann, Helmut Kortmann, Eric Verhoeven, Heiner Wenk

**Affiliations:** 1 Department for Vascular Medicine, Vascular Surgery, Endovascular Therapy and Angiology, University Heart and Vascular Center, University Medical Center Hamburg-Eppendorf, Martinistrasse 52, 20246 Hamburg, Germany; 2 Department for Surgery, Hospital Wilhelmsburg Groß-Sand, Groß-Sand 3, 21107 Hamburg, Germany; 3 Institute for Medical Biometry and Epidemology, University Medical Center Hamburg Eppendorf, Martinistrasse 52, 20246 Hamburg, Germany

**Keywords:** Aortic aneurysm, Abdominal, Incisional hernia, Onlay mesh, Fascial suture closure, Randomised controlled trial

## Abstract

The reported incidence of incisional hernia following repair of abdominal aortic aneurysm (AAA) via midline laparotomy is up to 69%. This prospective, multicenter, double-blind, randomised controlled trial was conducted at eleven hospitals in Germany. Patients aged 18 years or older undergoing elective AAA-repair via midline incision were randomly assigned using a computer-generated randomisation sequence to one of three groups for fascial closure: with long-term absorbable suture (MonoPlus^®^, group I), long-term absorbable suture and onlay mesh reinforcement (group II) or extra long-term absorbable suture (MonoMax^®^, group III). The primary endpoint was the incidence of incisional hernia within 24 months of follow-up, analysed by intention to treat. Physicians conducting the postoperative visits and the patients were blinded. Between February 2011 and July 2013, 104 patients (69.8 ± 7.7 years) were randomised, 99 of them received a study intervention. The rate of incisional hernia within 24 months was not significantly reduced with onlay mesh augmentation compared to primary suture (*p* = 0.290). Furthermore, the rate of incisional hernia did not differ significantly between fascial closure with slow and extra long-term absorbable suture (*p* = 0.111). Serious adverse events related to study intervention occurred in five patients (5.1%) from treatment *groups II* and *III*. Wound healing disorders were more frequently seen after onlay mesh implantation on the day of discharge (*p* = 0.010) and three (*p* = 0.009) and six (*p* = 0.023) months postoperatively. The existing evidence on prophylactic mesh augmentation in patients undergoing AAA-repair via midline laparotomy probably needs critical review. As the implementation of new RCTs is considered difficult due to the increasing number of endovascular AAA treated, registry studies could help to collect and evaluate data in cases of open AAA-repair. Comparisons between prophylactic mesh implantation and the small bite technique are also required. Trial registration: ClinicalTrials.gov Identifier: NCT01353443. Funding Sources: Aesculap AG, Tuttlingen, Germany.

## Introduction

After repair of abdominal aortic aneurysm (AAA) by midline laparotomy, presence of incisional hernia is reported in 12.4–69% of cases [1–5]. In a systematic review and meta-regression study the weighted mean rate of incisional hernia 23.8 months after abdominal midline incision was 12.8% [6]. Patient-related risk factors include increasing age, male gender, chronic obstructive pulmonary disease, obesity (body mass index > 30 kg/m^2^) or cachexia, smoking, diabetes and history or previous surgery for abdominal aortic aneurysm (AAA) [1, 6, 7]. In addition, technical conditions such as emergency surgery, off-midline incision and contaminated/dirty wounds increase the risk of developing an incisional hernia [8].

The combined rate of incisional hernia after AAA-repair was 20.1% in a systematic review including seven studies [9], but also incidences of up to 69% were reported in high-risk patients under long-term follow-up [5, 10]. The pathogenesis of AAA and abdominal wall herniation is complex and multifactorial. Dysregulation of connective tissue metabolism is assumed to be the cause of the clinical manifestation of an aneurysm or hernia [3, 11]. In addition to the potentially life-threatening risk of incarceration, persistent pain and disturbing cosmetic effects also significantly reduce the quality of life [12, 13]. Incisional hernia repair also represents a major economic burden for public health [14].

Prior to initiation of this study evidence for prophylactic non-absorbable mesh implantation in patients with AAA was limited. Since first description of this technique in elective AAA-repair [15], several prospective studies investigated prophylactic mesh application in high-risk patients [16–20], two of them in patients undergoing AAA-repair [16, 21]. In all studies mesh was used in sublay or preperitoneal position. However, safely creating a space between the rectus muscle and its posterior sheath can be difficult in some cases [22], especially for vascular surgeons being not routinely trained in this technique [23, 24]. Two prospective studies [25, 26] investigated the technically more simple onlay technique and reported no incisional herniation in 36 and 46 months of follow-up, respectively. However, both trials examined patients with gastrointestinal pathologies and did not enroll AAA-patients.

Therefore, this study was planned to primary investigate whether implantation of a non-absorbable prophylactic onlay mesh is superior to primary fascial suture with regard to development of incisional hernia within 24 months after AAA-repair via midline laparotomy. Secondary endpoints were safety, wound complications, pain, time-to-return to normal activities and working life, and quality of life. Another secondary endpoint was the non-inferiority of a new, ultra-long-term absorbable suture (MonoMax^®^) compared to a long-term absorbable suture (MonoPlus^®^) with respect to the incidence of incisional hernia. Although various randomised controlled trials (RCT) and meta-analyses comparing different sutures indicated that the use of a long-term absorbable monofilament suture in a running suture technique seemed to be the best choice for abdominal wall closure [1], at the time of study planning, experience with MonoMax was limited [27].

## Methods

### Trial design

This was a prospective, multicentre, double-blinded, parallel-group, balanced randomised [1:1:1] controlled trial. The final study protocol was approved by the ethics committee of the University of Hamburg and secondary approval was obtained from all local ethics committees responsible for the participating hospitals. The trial was performed according to the Consolidated Standards of Reporting Trials (CONSORT) Statement recommendations [28] and registered in the clinicaltrials.gov database (NCT01353443). There were no changes in methods after trial commencement.

### Participants and setting

All adults aged 18 years and older with an indication for elective treatment of AAA by median laparotomy were eligible to participate. Exclusion criteria were previous midline laparotomy, emergency surgery for AAA, life expectancy less than 2 years, current immunosuppressive therapy, coagulopathy, chemotherapy within the last 4 weeks, radiotherapy on the treated region within the last 2 months, participating in other investigational drug or medical device studies, pregnancy, and mental or social reasons affecting the ability to fulfil study requirements. All participants gave written informed consent. The trial was conducted in eleven German hospitals between February 2011 and July 2013 with 34 or 35 patients per group.

### Randomisation and blinding

Patients were assigned a unique computer-generated randomisation identification number, which determined their group membership. The computer-generated random numbers were calculated for all patients, i.e. there was no block randomisation. Patients were randomly allocated to receive either fascial closure with long-term absorbable suture (group I, MonoPlus), long-term absorbable suture and onlay-mesh reinforcement (group II, MonoPlus and mesh) or extra long-term absorbable, more flexible and elastically suture (group III, MonoMax).

Both the investigators conducting the postoperative visits and the patients were unaware of the method used for wound closure until the endpoint of the trial. To avoid bias, the surgeons who performed the laparotomy and fascial closure were not involved in follow-up of patients.

### Procedures

All vascular surgeons were trained on a pig model specifically in dissection of the ventral fascia, suturing the fascia as well as positioning and fixation the onlay mesh in exactly the same way to achieve comparable results. Fascial closure was always applied as a continuous suture anchored cranially and caudally to the incision (loop technique) with a suture to wound length ratio of 4:1 in accordance with the recommendations of the European hernia society [1]. In addition, the first operation was accompanied by a proctor in each centre.

In group I fascial closure was achieved by a long-term absorbable synthetic monofilament suture made of polydioxane (MonoPlus^®^, suture size USP 1, needle HRT 48, 150 cm loop, Aesculap AG, Tuttlingen, Germany).

In group II fascia was closed using the same suture material described above. Then a space 10 cm wide and adapted in length to the median laparotomy was created between the anterior rectus fascia and the subcutaneous fat tissue. A monofilament, light-weight, large-pored, polypropylene mesh (Optilene^®^ Mesh Elastic 10 × 35 cm, Aesculap AG, Tuttlingen, Germany) was then placed ventrally of the anterior rectus fascia (onlay position) with overlapping the incision 5 cm to the right and left and 2.5 cm cranially and caudally. Therefore, given the total mesh length of 35 cm, the maximum permitted length of the incision was 30 cm. A longer incision led to the exclusion from the study. For a shorter incision, the mesh was cut to match the incision length and dimensions of the mesh were documented. The mesh was fixed to the aponeurotic surface, avoiding excessive tension with interrupted stitches of a long-term absorbable suture material (MonoPlus, UPS 2/0, needle HR 26 needle, 70 cm, Aesculap AG, Tuttlingen, Germany). The stitch distance was about 5 cm and each stitch was about 1 cm from the edge of the mesh. For each stitch, 4–6 knots were used for anchoring.

In group III fascial closure was done by an extra long-term absorbable synthetic monofilament suture made of poly–4–hydroxybutyrate (MonoMax^®^, USP 1, needle HRT 48, 150 cm, Aesculap AG, Tuttlingen, Germany).

In all procedures the peritoneum was not generally sutured. A drainage was placed in the subcutaneous layer, no subcutaneous sutures were applied, and skin was closed with a stapler.

### Outcomes

Primary endpoint was the incidence of incisional hernia within 24 months of follow-up. Incisional hernia was defined as any abdominal wall gap with or without a bulge in the area of the postoperative scar perceptible or palpable by clinical examination or imaging, as determined by the European Hernia Society [29]. To measure this outcome, all patients were routinely invited to outpatient departments of the eleven hospitals and underwent physical examination of the abdomen and ultrasound scan of the aortic reconstruction and abdominal wall 3, 6, 12 and 24 months postoperatively. If ultrasound was inconclusive, an abdominal CT-scan was performed.

Secondary endpoints were the rate of incisional hernia within 12 months after surgery, non-inferiority of MonoMax compared to MonoPlus regarding the rate of incisional hernia (both assessed as described above), safety, postoperative wound complications (assessed clinically), postoperative pain (self-reported), time to return to normal activities and working life (self-reported) and quality of life (self-reported). Wound healing disorders were detected during visits by a vascular surgeon 2 days after surgery, on the day of discharge, 3, 6, 12 and 24 months postoperatively and were defined as infection (requiring antibiotic treatment), necrosis, fistula, wound dehiscence, hematoma or seroma. Serious adverse events were collected for the duration of the study and subjects were asked to report any events directly to study personnel. Furthermore, all patients were asked to fill out the questionnaire EuroQol five dimensions (EQ-5D) and indicate their pain on a visual analogue scale preoperatively, 2 days after surgery, on the day of discharge, 3, 6, 12 and 24 months postoperatively. No changes to trial objectives were implemented after the start of the study.

### Sample size

The clinical investigation sample size was calculated for the primary objective, assuming 30% incidence in the primary suture group and 10% incidence in the mesh group and chosen for 90% power and 0.05 two-sided significance level. We estimated five dropouts in each group, i.e. a total of 15 patients. Therefore, it was planned to enrol a total of 282 patients who met the inclusion criteria (94 patients per group). Unfortunately due to difficult patient recruitment, the trial was terminated in July 2013 for funding restrictions.

### Data management and statistical analysis

An independent Data Monitoring Committee reviewed data at regular intervals. All data were entered twice into an SPSS database (SPSS Statistics Version 23, IBM, Reference Number 1698495, March 2015). A statistical analysis plan was written and approved by the ethics committee before start of the analyses.

### Primary outcome

For incidence of incisional hernia an adjusted Cox regression was used with the type of reconstruction (no mesh vs. mesh implantation) and the type of suture material (MonoPlus vs. MonoMax) as fixed factors. Age and gender served as covariates. Since this was a superiority study, the primary analysis was performed in the intention-to-treat collective and two-sided at significance level *α* = 0.05. The null hypothesis was rejected if the p-value of the factor "type of reconstruction" was less than 0.05. The three intervention groups were presented using Kaplan–Meier plots. As sensitivity analyses, a Cox regression adjusted for covariates (baseline characteristics) unequally distributed between the groups (daily number of cigarettes, the presence of cardiovascular diseases, malignancies and previous surgery) were performed (indicator was a *p*-value from ≤ 0.2). Typical risk factors of incisional hernia such as smoking, body mass index elevation and COPD were equally distributed between the groups, therefore no adjustment was made using these variables.

### Secondary outcome

The incidence of incisional hernia within 12 months after onlay mesh (group II) compared to no mesh (group I and III) was determined using a Kaplan–Meier plot and absolute and relative frequencies at different points in time.

For the non-inferiority of the suture material MonoMax (group III) compared to MonoPlus (group II) with respect to the rate of incisional hernia 3, 6, 12 and 24 months postoperatively, a difference in the rate of incisional hernia of 5% absolute was defined. A Cox regression model was used and MonoMax was estimated to be inferior if the upper limit of the two-sided 95% confidence interval (CI) for the difference in the rate of incisional hernia (MonoMax–MonoPlus) was below 5%.

For the comparison of categorical variables, including (severe) adverse events, Chi^2^-tests or Fisher's exact tests (in case of expected frequencies < 5) were used, separately for individual points in time. All secondary and sensitivity analyses were performed exploratively and the *p*-values were used as descriptive parameters.

The average time in days to return to activities of normal or working life was analysed using the same Cox regression model as in the primary analysis. For the differences in general health-related quality of life (EQ-5D) and postoperative pain a linear regression was performed. Changes to the baseline were considered and additionally adjusted for the baseline value.

## Results

**Fig. 1:**
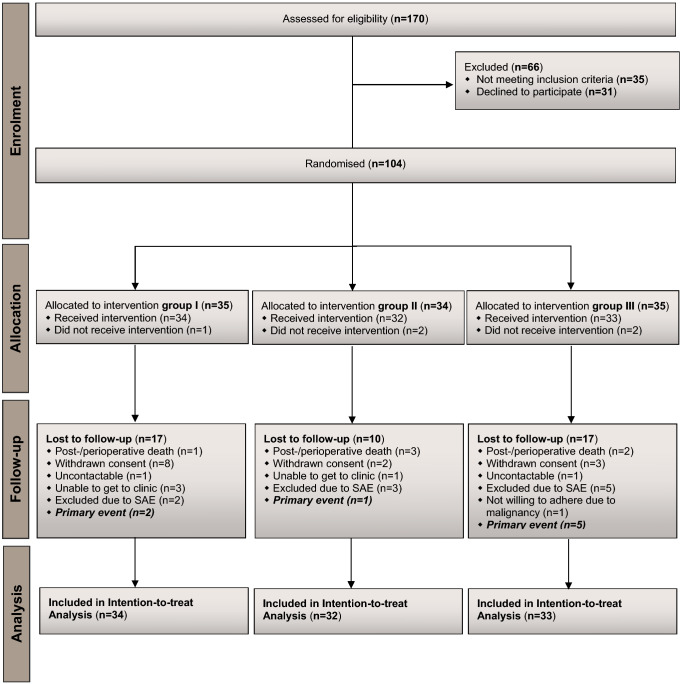
CONSORT flow diagram of the trial

Between February 2011 and July 2013, a total of 170 patients undergoing AAA-repair via midline laparotomy were examined for their suitability. Thirty-five patients met at least one of the exclusion criteria and 31 patients declined to participate. The remaining 104 patients with a mean age of 69.3 (range 47.6) years were assigned to one of the three groups. Four patients (one in group I, two in group II and one in group III) did not receive allocated intervention due to an incision length of median laparotomy over 30 cm. Another patient, randomised in group III, died before study intervention due to an intraoperative myocardial infarction. The other 99 patients were treated according to study protocol. The mean (range) duration of follow-up was 814 (2–1015) days, and 44 (48.89%) patients were lost for follow-up during the study, 17 of whom were assigned to fascial closure with MonoPlus, 10 to onlay mesh reinforcement and 17 to fascial closure with MonoMax. Eight (18.18%) of 44 patients with incomplete follow-up had a primary event before discontinuation (Fig. 1).

### Baseline characteristics

Descriptions of patients’ characteristics at baseline are given in Table 1.

**Table 1:** Baseline characteristics

	Total (*n* = 104)	Group I (*n* = 35)	Group II (*n* = 34)	Group III (*n* = 35)	*p* value
**Male**	**94 (90.38%)**	**32 (91.4%)**	**33 (97.1%)**	**29 (82.9%)**	**0.151**^**b**^
**Female**	**10 (9.62%)**	**3 (8.6%)**	**1 (2.9%)**	**6 (17.1%)**	
Age (years)	69.29 (8.15)	67.37 (9.55)	70.53 (7.80)	70.20 (6.69)	0.208^c^
BMI (kg/m^2^)*	103 (99.04%)	26.82 (2.94)	26.58 (4.04)	27.74 (4.82)	0.447^c^
BMI > 30 kg/m^2^	22 (21.15%)	6 (17.1)	7 (20.6)	9 (25.7)	0.677^a^
Smoker	49 (47.12%)	13 (38.2%)	20 (58.8%)	16 (45.7%)	0.227^a^
Daily number of cigarettes**	43 (41.35%)*	**18 (9.03)**	**19 (14.49)**	**12 (5.94)**	**0.200**^**c**^
Alcohol consumption	68 (65.38%)	22 (62.9%)	23 (69.7%)	23 (69.7%)	0.784^a^
Frequency of alcohol consumption					
Less than once weekly	15 (14.42%)	5 (22.7%)	4 (17.4%)	6 (26.1%)	0.488^b^
Once weekly	18 (17.31%)	8 (36.4%)	4 (17.4%)	6 (26.1%)	
2–6 weekly	23 (22.12%)	4 (18.2%)	11 (47.8%)	8 (34.8%)	
Daily	12 (11.54%)	5 (22.7%)	4 (17.4%)	3 (13.0)	
Insulin-dependent diabetes	4 (3.85%)	2 (5.9%)	0 (0.0%)	2 (5.9%)	0.542^b^
COPD	16 (15.38%)	7 (20.0%)	4 (12.5%)	5 (15.2%)	0.696^a^
ASA classification					1.000^b^
II	41 (39.43%)	14 (40.0%)	14 (41.2%)	13 (38.2%)	
III	61 (58.65%)	20 (57.1%)	20 (58.8%)	21 (61.8%)	
IV	1 (0.96%)	1 (2.9%)	0 (0.0%)	0 (0.0%)	
Unspecified	1 (0.96%)	0 (0.0%)	0 (0.0%)	1 (2.9%)	
**Cardiovascular diseases**	**65 (62.50%)**	**27 (77.1%)**	**17 (51.5%)**	**21 (61.8%)**	**0.086**^**a**^
Pulmonary diseases	15 (14.42%)	7 (20.6%)	3 (9.1%)	5 (14.7%)	0.417^a^
Gastrointestinal diseases	11 (10.58%)	4 (11.4%)	3 (9.4%)	4 (11.8%)	1.000^b^
Diseases of the urinary tract	12 (11.54%)	3 (8.6%)	6 (18.2%)	3 (8.8%)	0.474^b^
Hemato-oncologic diseases	3 (2.88%)	1 (2.9%)	1 (3.0%)	1 (2.9%)	1.000^b^
Endocrine diseases	23 (22.12%)	8 (22.9%)	8 (24.2%)	7 (20.6%)	0.937^a^
Immune mediated diseases	1 (0.96%)	1 (2.9%)	0 (0.0%)	0 (0.0%)	1.000^b^
Skin diseases	6 (5.77%)	2 (5.7%)	3 (9.1%)	1 (2.9%)	0.522^b^
Disorders of muscle, skeleton and connective tissue	9 (8.65%)	2 (5.9%)	2 (6.1%)	5 (14.7%)	0.499^b^
**Malignancies**	**7 (6.73%)**	**0 (0.0%)**	**5 (15.6%)**	**2 (5.9%)**	**0.030**^**b**^
Previous surgery***	**84 (80.77%)**	**31 (88.6%)**	**29 (85.3%)**	**24 (68.6%)**	**0.075**^**a**^
Further diseases influencing wound healing	2 (1.92%)	1 (3.1%)	1 (3.2%)	0 (0.0%)	0.544^b^

Data are number of patients (%) or mean (SD). Bold indicates test for even distribution between the groups by Chi^2^ test (a), Fisher’s exact test (b) or One-way ANOVA (c). Indicator for unequally distributed baseline characteristics was a *p* value from ≤ .2

*ASA* American Society of Anesthesiologists, *BMI* body mass index, *COPD* chronic obstructive pulmonary disease

*Number of patients who submitted a statement to this effect is 103

**Number of patients who submitted a statement to this effect is 43

***Including 32 abdominal operations via approaches other than median laparotomy

### Primary endpoint

**Fig. 2:**
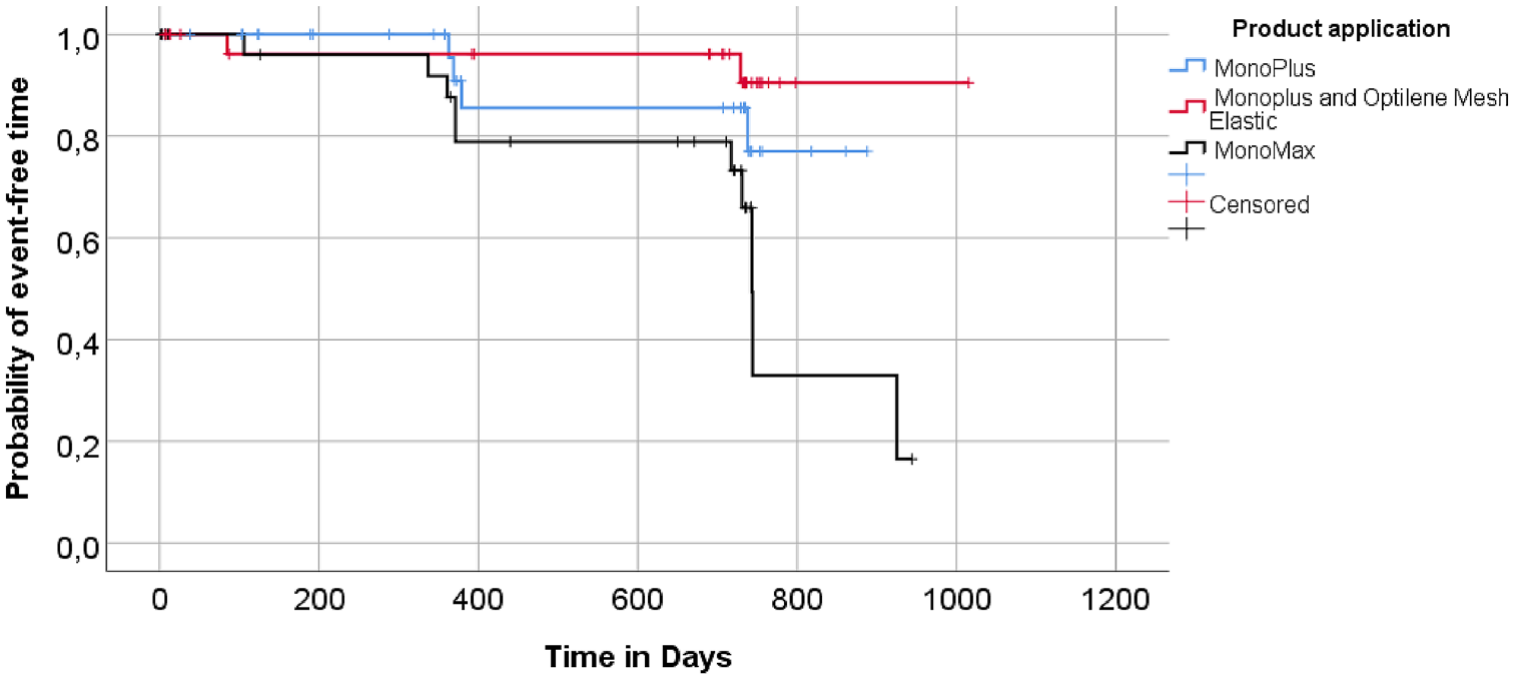
Kaplan–Meier curve for the probability of an event-free time. Comparison of the three treatment groups MonoPlus (group I), Monoplus and Olay Mesh Reinforcement (group II) and MonoMax (group III) based on the primary event “incision hernia within 24 months”

16 (16.2%) of 99 patients developed incisional hernia during 24 months of follow-up, 4/34 (11.8%) in group I, 2/32 (6.25%) in group II, and 10/33 (30.3%) in group III. Non-implantation of onlay mesh (group I and III) showed a 2.52-fold higher risk (hazard) of developing an incisional hernia within 24 months compared to onlay mesh reinforcement (group II), but a superiority of onlay mesh implantation over primary suture could not be statistically proven (*p* = 0.290). Fascial closure with MonoMax (group III) had a 2.67-fold higher risk (hazard) of developing an incisional hernia within 24 months than with MonoPlus (group I and II), but again without statistical significance (*p* = 0.111). The adjustment for the covariates age and gender also had no influence on these findings. The Kaplan–Meier curve for the probability of an event-free time is illustrated in Fig. 2.

The sensitivity analysis showed similar results except that treatment with MonoPlus and onlay mesh augmentation (group II) showed a tendentially lower rate of incisional hernia at 24 months than after fascial closure with Monoplus (group III) (*p* = 0.018, Table 2).

**Table 2:** Incidence of incisional hernia in all patients included in intention-to-treat analysis

	Rate of primary event (%)	Hazard ratio (95% CI)	*p* value
Adjusted cox-regression			
Onlay mesh (group II) vs no onlay mesh (group I and III)	2/32 (6.25) vs 14/67 (20.90)	2.519 (0.455; 13.939)	0.290
MonoPlus (group I and group II) vs Monomax (group III)	6/66 (9.09) vs 10/33 (30.30)	2.666 (0.797; 8.912)	0.111
Male vs female	15/94 (15.96) vs 1/10 (10.00)	0.498 (0.063; 3.961)	0.510
Age		1.171 (0.558; 2.457)	0.677
Sensitivity analysis			
MonoPlus (group I) vs Monoplus and onlay mesh (group II)	4/34 (11.76) vs 2/32 (6.25)	2.422 (0.442; 13.281)	0.308
MonoMax (group III) vs Monoplus and onlay mesh (group II)	10/33 (30.30) vs 2/32 (6.25)	6.364 (1.379; 29.376)	**0.018**
Male vs female	15/94 (15.96) vs 1/10 (10.00)	0.342 (0.032; 3.607)	0.372
Age (unit = 10 years)		0.841 (0.407; 2.405)	0.737
Daily number of cigarettes (unit = 1 cigarette)		0.965 (0.059; 1.587)	0.249
Cardiovascular disease (no vs yes)	5/37 (13.51) vs 11/65 (16.92)	0.923 (0.250; 3.402)	0.904
Malignancy (no vs yes)	15/93 (16.13) vs 0/7 (0.00)	Incalculable	0.995
Previous surgery (no vs yes)	4/20 (20.00) vs 12/84 (14.29)	1.992 (0.463; 8.576)	0.355

Calculated using a Cox regression model with age and gender as covariates. Also sensitivity analyses with a Cox regression model adjusted for baseline characteristics (covariates) unequally distributed between the groups (indicator was a *p*-value from ≤ 0.2)

*ITT* intention to treat, *CI* confidence interval

### Rate of incisional hernias after 12 months

**Fig. 3:**
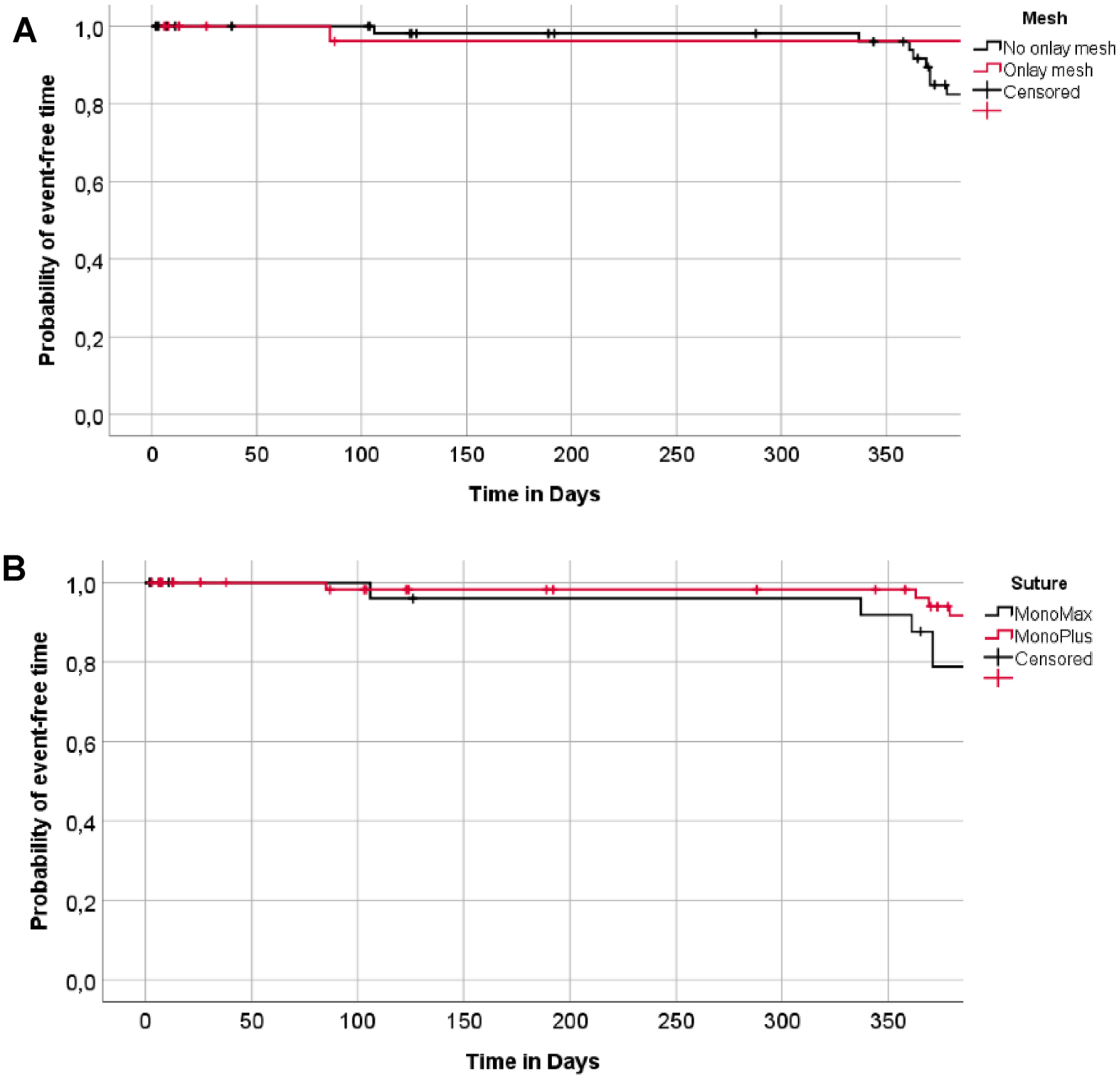
Kaplan–Meier curve for the probability of an event-free time. Comparison of onlay mesh reinforcement (group II) versus no onlay mesh reinforcement (group I and III) **a** and of MonoPlus (group I and II) versus MonoMax (group III) **b** in regards to the secondary event “incision hernia within 12 months”

The adjusted Cox model showed a tendentially higher rate of incisional hernia following onlay mesh augmentation (group II) compared to sutured fascial closure (group I and III) (HR = 1.87, *p* = 0.500) in the first 12 months postoperatively. In the following 12 months this tendency reversed in favour of onlay mesh. Furthermore, no relevant difference was found between MonoPlus (group I and II) and MonoMax (group III) regarding the occurrence of incisional hernia within 12 months (HR = 1.46, *p* = 0.615) (Fig. 3).

### Comparison of MonoMax versus MonoPlus

Since the upper limit of the CI of the risk difference between both sutures 3, 6, 12 and 24 months postoperatively was never below 8.88%, the non-inferiority of MonoMax compared to MonoPlus was never met.

### Morbidity and mortality

Six of 99 (6.06%) patients died due to pulmonary insufficiency in pneumonia (group I), pneumococcal sepsis (group II), malignancy (group III) and cardiac failure (group II *n* = 2; group III *n* = 1). Another patient died after randomisation and before receiving study intervention due to myocardial infarction.

Serious adverse events associated with study intervention occurred in five (5.05%) and unrelated to study intervention in 28 (28.28%) patients (Table 3).

**Table 3:** Serious adverse events

Complication	Treatment	Study group
With relation to study intervention (*n* = 5)		
Fascial dehiscence with bowel incarceration (*n* = 1)	Relaparotomy, bowel resection	II
Symptomatic incisional hernia (*n* = 2)	Relaparotomy, mesh implantation	III, III
Infected seroma* (*n* = 2)	Surgical revision, irrigation, mesh removal (*n* = 1), antibiosis	II, II
Without relation to study intervention		
Abdominal		
Bleeding from aortic anastomosis (*n* = 1)	Relaparotomy, suture	
Partial occlusion of aorto-iliac graft (*n* = 1)	Relaparotomy, repeat aorto-bi-iliac reconstruction	
Bride ileus (*n* = 1)	Relaparotomy, adhesiolysis	
Rectosigmoid ischemia (*n* = 1)	Relaparotomy, bowel resection	
Bowel paralysis (*n* = 1)	Pharmaceutical	
Malpositioned urethral catheter with urethral injury (*n* = 1)	Cystoscopy, bladder irrigation via transurethral catheter	
Enteritis (*n* = 2)	Saline hydration, pharmaceutical	
Colitis (*n* = 1)	Antibiosis	
Pulmonary (*n* = 3)		
Pneumonia (*n* = 2)	Antibiosis	
Peripheral pulmonary artery embolism due to deep vein thrombosis (*n* = 1)	Anticoagulation	
Renal (*n* = 3)		
Worsening of pre-existing kidney dysfunction** (*n* = 3)	Saline hydration, pharmaceutical	
Cardiologic (*n* = 4)		
Myocardial infarction (*n* = 2)	Pharmaceutical	
Cardiac arrest (*n* = 1)	CPR, intensive care management	
3rd degree AV block (*n* = 1)	Pacemaker implantation	
Neurologic (*n* = 1)		
Postoperative delirium (*n* = 1)	Pharmaceutical	
Others (*n* = 2)		
Catheter infection (*n* = 1)	Antibiosis	
Skin eczema (*n* = 1)	Pharmaceutical	
Disease of femoro-popliteal arteries (*n* = 3)		
Stage III POD (*n* = 1)	Surgical revascularization	
Embolizing popliteal aneurysm (*n* = 1)	Femoro-popliteal bypass, secondary thigh amputation	
Femoro-popliteal graft infection (*n* = 1)	Antibiosis	

*Required mesh removal in one case

**Without necessity of dialysis

*CPR* cardiopulmonary resuscitation, *POD* peripheral occlusive disease

Wound healing disorders were more frequently seen after onlay mesh implantation (group II) on the day of discharge (*p* = 0.010) and three (*p* = 0.009) and six (*p* = 0.023) months postoperatively. This result was based on six seromas detected exclusively in group II on the day of discharge (*p* = 0.102) and after 3 months (*p* = 0.001). Two seromas required surgical revision due to superinfection, one of which led to removal of the onlay mesh (Table 3).

### Quality of life and pain

After adjusted Cox regression, no relevant differences were found between onlay mesh (group II) and primary suture (group I and III) (*p* = 0.780) as well as MonoPlus (group I and II) and MonoMax (group III) (*p* = 0.390) with respect to activities of daily life. Similar results were found when comparing mesh and no mesh (*p* = 0.590) and the two suture materials (*p* = 0.650) in terms of return to work. Furthermore, at no time differences were found between treatment groups regarding EQ-5D scores and postoperative pain.

## Discussion

### Primary endpoint

In the current study (AIDA), the rate of incisional hernia 24 months after AAA-repair by midline laparotomy was not significantly different between onlay mesh application and sutured fascial closure (*p* = 0.290; HR = 2.52) and between primary suture with MonoMax and MonoPlus (HR = 2.67; *p* = 0.111).

A recently published meta-analysis of four RCTs comparing prophylactic mesh implantation with primary suturing of the abdominal wall in patients undergoing AAA-repair via midline laparotomy showed in a pooled analysis that mesh reinforcement significantly reduces the risk of incisional hernia compared to standard suture closure [30]. The only included RCT examining a non-absorbable mesh in onlay position (PRIMA trial) showed a significantly reduced rate of incisional hernia in high risk patients [24]. Based on these data, prophylactic mesh reinforcement is recommended in several guidelines in high risk patients undergoing AAA-repair via midline laparotomy [31, 32]. However, there are some methodical differences between the PRIMA and the AIDA trial, which may explain different incidences of incisional hernia in those studies.

First, 100% of the follow-up examinations in the AIDA trial included sectional imaging (abdominal ultrasound or CT-scan), which is a more accurate procedure for detecting incisional hernia compared to clinical examination alone. In the PRIMA trial, only 59% of patients underwent radiological examination [24]. In addition, 330 of the 480 patients in the PRIMA trial had a body mass index of 27 kg/m^2^ or higher [24] and, especially in obese patients, the clinical examination of the abdomen appears to be flawed with regard to the detection of incisional hernia. This might have led to an underestimation of incisional hernia during physical examination across all groups. This assumption is supported by previous studies describing rates of incisional hernia of 0% when non-systematic imaging was performed [23, 25]. In the AIDA study, where obesity was not a specific inclusion criteria and the body mass index was balanced across all groups (body mass index [kg/m^2^] − mean (SD): group I 26.82 (2.94); group II 26.58 (4.04); group III 27.74 (4.82); *p* = 0.447), a total of 16 out of 99 patients were found to have an incisional hernia 24 months postoperatively. Addressing the problem of limited clinical evaluability, the European Hernia Society recommends in its guideline that prospective studies with incisional hernia as primary outcome integrate either dynamic ultrasound or CT-scan in their follow-up [1].

Second, we exclusively investigated patients undergoing AAA-repair. Although the subgroup analysis of AAA-patients of the PRIMA trial showed a significantly lower rate of incisional hernia with onlay mesh (10/61, 16%) in contrast to primary suture (16/37; 43%), it must be noted that in this subgroup the rate of incisional hernia after primary suture was markedly increased compared to those in obese patients and the overall collective, which might have influenced the good result of onlay mesh augmentation in AAA patients [24]. Whether the occurrence of AAA has a greater impact on the primary development of incisional hernia than obesity is not known. However, Burger et al. were able to prove that AAA (*p* = 0.010), in contrast to obesity, is an independent risk factor for recurrence of incisional hernia [7].

Third, the question arises to what extent the type of fascial suture with or without additional mesh augmentation has an influence on the comparability of previous RCTs [17, 23, 24, 33]. The recommended suture to wound length ratio of 4:1 was met in all RCTs, the recommended closure with long-term absorbable sutures in continuous technique in three of four [1]. However, whether the small or large bite technique was used was not reported in any of the trials. Previous studies could demonstrate a significantly reduced rate of incisional hernia using the small bite technique, taking bites of 5–8 mm of the fascia and stitches every 5 mm, compared to the large bite technique, taking bites of 10 mm of the fascia and stitches every 10 mm [34, 35]. The use of the large bite technique in the AIDA study, even if standardized and uniformly performed, might have contributed to an increased rate of incisional hernia across all groups. To what extent an onlay mesh reinforcement in addition to the small bite technique has a preventive effect on the development of incisional hernia is subject of current research [36].

Finally, regardless of all of reasons mentioned above, it must be emphasized that the AIDA trial was underpowered with regard to the primary endpoint. This may have caused the trend towards superiority of onlay mesh but ultimately without statistical significance.

### Secondary outcomes

In the first 12 months of follow-up, patients treated with mesh showed tendentially, but not significantly more incisional hernia than those treated with primary suture (HR = 1.87, *p* = 0.500). Since the ratio of incisional hernias thereafter reverses to the disadvantage of patients treated with suture closure, it can be concluded that incisional hernias occur earlier but not more frequently after mesh than after primary suture. With the exception of a possibly more extensive ultrasound examination in patients with onlay mesh implantation, there seems to be no objective reason for an earlier manifestation or detection of incisional hernia in this group.

The non-inferiority of the suture material MonoMax compared to MonoPlus could not be proven at 3, 6, 12 and 24 months postoperatively. Due to its increased absorption and degradation period, its high elasticity and its long-lasting linear tensile strength retention, MonoMax was thought to be able to reduce the rate of incisional hernia, as increased suture tension and the decrease in initial strength of the abdominal fascia over time were thought to play an important role in the development of incisional hernia [21, 37]. Its safety and efficacy has already been demonstrated the prospective controlled ISSAAC-trial [37]. Although the observed rate of incisional hernia of 14.0% in the ISSAAC-study was lower than that of the historical control group (21.3%), this single-arm trial was not designed for a primary comparison of the two distinct study populations in terms of the rate of incisional hernia, so that methodological limitations narrow the evidence regarding this endpoint [37]. However, a direct comparison of MonoMax vs. MonoPlus in a randomised setting does not show an advantage of MonoMax.

In terms of safety, the rate of wound healing disturbances after onlay mesh implantation (group II) was significantly increased compared to primary suture (group I and III) at discharge and three and 6 months postoperatively. This was due to the significantly more frequent occurrence of seromas after mesh application, which is a well-known problem [19, 24], especially in onlay position [24, 38] and usually causes no discomfort. However, despite routine insertion of a subcutaneous drainage, the rate of seroma after mesh implantation was remarkable with 18.75% (6/32) in this trial. As in the case of incisional hernia, this is most likely due to the better detection by consistently performed sectional image diagnosis. Another influence might have been the fixation of the mesh by sutures, although fixation by fibrin did not lead to an expected reduction of the seroma rate in the past [24]. With values of body mass index between 22.7 and 37.1 kg/m^2^, no tendency towards cachexia or obesity with regard to the occurrence of seroma could be detected in this trial.

For all subjectively assessed characteristics, such as postoperative pain, time to return to normal activities and working life, and quality of life, no relevant difference was found between the three groups. This underlines our opinion that onlay mesh implantation is a simple and quick method, which feels no different to a primary suture for the patient immediately after the operation, and during follow-up. It also implies that seromas were not perceived as more painful. Our results of postoperative pain and quality of life depending on the type of abdominal wall closure are in consent with those others [24].

## Limitations

The main limitation and reason for relativizing of our results was the below-average sample size of each group as a consequence of an increasing preference for endovascular techniques in AAA treatment during the study period, which, as in previous studies [23], led to difficulties in patient recruitment.

## Conclusion

Since the incidence of incisional hernia was not significantly different between mesh application and primary suture, the existing evidence and corresponding guideline recommendations [31, 32] for prophylactic mesh augmentation in patients undergoing open AAA-repair should be critically reviewed. The occurrence of seromas is a common observation after mesh implantation, whereby the type of mesh fixation, use of subcutaneous drainage and body mass index do not seem to have any influence on this. The implementation of new RCTs investigating prophylactic mesh reinforcement in AAA-patients must be considered to be difficult due to the increasing number of endovascular procedures. Registry studies could help to collect and analyse data in cases where AAA are treated by open surgery. In addition, the results of a currently ongoing comparative study between prophylactic onlay mesh augmentation and small bite fascial closure must be awaited and implemented in future practice recommendations.
